# The pattern of neonatal admissions and mortality at a regional and district hospital in the Upper West Region of Ghana; a cross sectional study

**DOI:** 10.1371/journal.pone.0232406

**Published:** 2020-05-04

**Authors:** Edem M. A. Tette, Edmund T. Nartey, Benjamin D. Nuertey, Emmanuel A. Azusong, Dominic Akaateba, Judith Yirifere, Augustine Alandu, Nana Ayegua Hagan Seneadza, Naa Barnabas Gandau, Lorna A. Renner

**Affiliations:** 1 Department of Community Health, University of Ghana Medical School, Accra, Ghana; 2 Centre for Tropical Clinical Pharmacology and Therapeutics, University of Ghana Medical School, Accra, Ghana; 3 Public Health Department, Tamale Teaching Hospital, Tamale, Ghana; 4 Upper West Regional Hospital, Wa, Ghana; 5 St. Joseph’s Hospital, Jirapa, Ghana; 6 School of Medical Sciences, University for Development Studies, Tamale, Ghana; 7 Department of Child Health, University of Ghana Medical School, Accra, Ghana; University of Michigan Medical School, UNITED STATES

## Abstract

**Background:**

High global neonatal deaths have triggered efforts to improve facility-based care. However, the outcomes achievable at different levels of care are unclear. This study compared morbidity and mortality patterns of newborns admitted to a regional and a district hospital in Ghana to determine outcome, risk and modifiable factors associated with mortality.

**Objective:**

This study compared morbidity and mortality patterns of newborns admitted to a regional and a district hospital in Ghana to determine outcome, risk and modifiable factors associated with mortality

**Methods:**

A cross-sectional study involving a records-review over one year at the Upper West Regional Hospital, and three years at St Joseph’s District Hospital, Jirapa was carried out. Age, sex, gestational age, weight, duration of admission, diagnosis, among others were examined. The data were analysed and statistical inference made.

**Results:**

Altogether, 2004 newborns were examined, comprising 1,241(62%) from St Joseph’s District Hospital and 763(38%) from Upper West Regional Hospital. The proportion of neonatal deaths was similar, 8.94% (St Joseph’s District Hospital) and 8.91% (Upper West Regional Hospital). Prematurity, neonatal sepsis, birth asphyxia, low birth weight, neonatal jaundice and pneumonia contributed the most to mortality and suspected infections including malaria accounted for almost half (45.5%). Mortality was significantly associated with duration of stay of 48 hours, being premature, and being younger than 3 days.

**Conclusion:**

Majority of the mortality among the neonates admitted was due to preventable causes. Better stabilization and further studies on the epidemiology of sepsis, prematurity, low birth weight, including the contribution of malaria to these and outcome of transferred neonates are needed.

## Background

Neonatal mortality contributes substantially to child mortality and has assumed great significance in recent times [1, 2]. An earlier report indicated that out of the 130 million babies born each year, approximately 4 million died within the first four weeks of life [1]. At the time, neonatal mortality accounted for about 38% of deaths in under-fives with the majority of these deaths occurring in low and lower middle-income countries. More recent reports have shown that, while mortality from older children reduced considerably, neonatal mortality only reduced slightly in many countries in response to the efforts made to achieve the Millennium Development Goal 4 [2,3]. This goal aimed at reducing by two thirds, between 1990 and 2015, the under-five mortality rate [2]. Thus, neonatal mortality is currently the major cause of child mortality [2, 3]. According to the Ghana Demographic and Health Survey 2014, neonatal mortality decreased from 30 to 29 per 1000 live births during the 15 years preceding the survey [3]. Since this reduction was at a slower pace than that of the infant and child mortality rates, the contribution of neonatal mortality to under-5 mortality rose from 28% to 48% over the same period. Consequently, in order to reduce child mortality further, significant reductions in neonatal mortality need to occur [1].

The causes of neonatal mortality are many. They include neonatal sepsis, other infections, low birth weight, prematurity, birth asphyxia, birth trauma, congenital malformations, neonatal tetanus, diarrhoea, and pneumonia, among others [1, 4–7]. Neonatal deaths are also related to the quality of care a woman receives during pregnancy and labour as well as the quality of services available for the care of newborns in health facilities [1, 8]. One of the interventions to promote the survival of newborns nation-wide is to create facilities for specialized neonatal care in hospitals, however, attention has been drawn to the need to improve these services [9]. Additionally, it is unclear whether this has been implemented uniformly across the country, as facility-based studies in Ghana are limited, creating a need to examine neonatal care in different settings. Such studies will provide information on the outcomes that are achievable at the different levels of care and highlight modifiable factors that can improve outcome.

District hospitals are located in district capitals and provide clinical care to geographically defined catchment areas with an average population size of 100,000 to 200,000 people. It is the first referral hospital in the health system of Ghana and receives referrals from health centres and community-based health planning and services (CHPS) compounds [10,11]. They normally have 50 or more beds and are run by the Government, mission or faith-based organisation. Medical officers provide clinical care as they usually lack specialists though some district hospitals have specialist particularly the faith-based ones. A study has shown that there are about 20 district hospitals to one regional or teaching hospital; they deliver 52% of all institutional deliveries, treat two-thirds of all obstetric complications and refer about 9% though individual variations in their ability to provide services exist [11]. Regional hospitals are secondary level facilities located in regional capitals and are mainly run by the government through the Ghana Health Service. They are larger units and provide specialized care to populations of about 1.2 million. They usually have specialist in all the major medical disciplines, Surgery, Medicine, Obstetrics & Gynaecology, and Paediatrics and have specialist nurses, for instance, in Ophthalmology and Ear, Nose & Throat (ENT). They serve as referral sites for patients seen at district hospitals. Studies on neonatal mortality in teaching hospitals in large cities were available at the time of the study but very little was known about neonatal mortality in district or regional hospitals and in more rural settings that was why we opted to this study at these sites. In addition, the Upper West Region had the highest regional neonatal mortality rate at 37 per1000 live births per year compared to the Northern and Upper East Regions which share its borders and had the lowest rates at 24 per1000 live births per year each in the 2014 Demographic and Health Survey [3].

The Upper West Region is one of the most deprived regions in the country. During the 2010 Population and Housing census, it had a population of 702,110 people and it was the most rural region in Ghana with only 16.3% of its population living in urban areas at the time [12]. Paediatric services are limited there and are mostly covered by general medical officers. There was no Paediatrician at the Upper West Regional Hospital (UWRH) at the time of the study however a neonatal unit had been set up in 2016 after the one at St Joseph’s Hospital (SJH) district hospital in Jirapa which was set up in 2015 and the first in the region. We examined the pattern of neonatal morbidity and mortality at the Upper West Regional Hospital which had recently established a neonatal unit and St Joseph’s Hospital, Jirapa, a district hospital with an established neonatal unit, in order to determine risk factors and modifiable factors associated with mortality, compare outcomes and identify measures that can improve outcome in this and similar settings.

## Materials and methods

### Ethical clearance

Permission to carry out this research was obtained from the Medical Directors of the Regional Hospital and St Joseph’s District Hospital. Ethical Approval was obtained from the Ghana Health Service Ethical Review Committee, Protocol ID No: GHS-ERC 09/03/17. The record review involved a review of patient’s records and as there was no contact with patients, we did not obtain consent from the participants so consent forms were not administered. This was part of the ethical clearance statement and permission was granted by the Ethical Review Committee. Also, the data were anonymized so that the final results could not be linked to individual patients.

### Study area

#### The Upper West Regional Hospital (UWRH), Wa

The Upper West Regional Hospital currently, performs a dual role as a municipal and regional hospital. It is also the main referral center within the healthcare delivery system in the Upper West Region and beyond. It has several departments, including clinical care, public health, nursing administration, financial management, pharmaceutical services and a health administration and support services (HASS) division. The hospital had 48 medical doctors consisting of 37 Ghanaian Doctors (including 3 specialists) and 11 Expatriate Doctors (including 9 specialists) at the end of 2016. Additionally, there were 222 nurses, 182 paramedical staff and a bed complement of 200 beds, spread over 9 wards. The hospital’s neonatal unit became functional during the latter part of 2016. The unit functions as a special care babies unit as it does not provide intensive care services such as mechanical ventilation, total parenteral nutrition or blood gas analysis. Neonatal admissions recorded from August to December 2016 were 248 with 39 recorded deaths (15.7%). The number of deliveries were 4,881 in 2014, 4,497 in 2015 and 4,915 in 2016.

#### St. Joseph’s Hospital (SJH), Jirapa

St. Joseph’s Hospital, SJH, assumes the role of a district hospital for the Jirapa District. It has seven (7) wards and this includes a neonatal unit, which started operations in 2015. The hospital provides 24hr outpatient services as well as other specialized care services. At the end of 2016 there were three (3) Medical Doctors, one hundred and forty-three (143) nurses and one hundred and three (103) paramedical staff. The bed capacity at the end of 2016 was 193, spread over 7 wards. The total deliveries for the year 2016 was 1,709. The hospital receives referral from all the health centres, CHPS (Community-Based Health Planning and Services) compounds and some of the district hospitals in the region. This accounts for the high delivery rates. The neonatal unit started providing services for patients from 2015 to date, but does not provide mechanical ventilation, total parenteral nutrition or blood gas analysis. The total number of admissions for 2016 was 446 with 52 deaths. This neonatal unit was the first to be established in the Upper West Region. Prior to the establishment of a unit at the UWRH, it was the major facility for neonatal referrals in the region.

### Study design and sampling

The study was a descriptive study with a cross sectional design. A records review and analysis of neonatal admissions and deaths occurring at the Upper West Regional Hospital, was carried out from 1^st^ January 2017 to 31^st^ December 2017 (1 year) and at St Joseph’s District Hospital from 1st January 2015 to 31^st^ December 2017 (3 years). Convenient sampling was used, consequently, consecutive admissions were enrolled during the study. We used the IMRaD format for reporting this study.

### Study population

Neonates admitted to the neonatal units of the hospitals and those who died in the first month of life in these units over the period from 1^st^ January, 2015 to 31^st^ December, 2017 for SJH and 1^st^January, 2017 to 31^st^ December, 2017 for the UWRH were included in the study.

### Data collection methods and instruments

Records of consecutive patients admitted to the neonatal units were examined. Thus, the sample size included all neonates admitted to the hospital during the study period. The time periods for data collection between the UWRH and the SJH District hospital were different because of the differences in times of operation. Whereas SJH has had a neonatal unit since 2015 and data were available from 2015 to 2017 (three years), the UWRH’s neonatal unit was set up in August 2016 and complete data on neonatal admissions were available for only the year 2017 (one year).

The data were obtained from computerised records containing summaries of neonatal admissions and deaths at SJH and from the neonatal admissions and discharge registers of the UWRH. Hand-written records from the latter were entered into a record form using a neonatal data extraction form to obtain the data from UWRH. These sources of data provided information on the child’s age, sex, birth weight, gestational age, diagnosis, date of admission and discharge, outcome, the diagnosis at admission and place of residence. Prematurity referred to a gestational age of less than 37 completed weeks. Neonatal deaths in this study refer only to deaths occurring in neonates admitted to these two hospitals during the study period and before discharge. It does not include any deaths occurring post-discharge in the community. The information in these registers were reported to have been obtained from case notes and some case notes were used to cross check some of the information collected when necessary.

### Data analysis

Every enrolled patient was allocated a unique number in this study which was used in the storage and management of all data relating to the patient. Neonatal data were captured and analyzed using Statistical Package for Social Sciences (SPSS) version 16.0. Cleaning of data was done using standardized queries to conduct range and logic check. Discrepant entries were rectified by reviewing the record form and other records as necessary. The programme was used to compute frequencies, proportions and means of study variables to produce tabular and graphical representation of the data. Comparisons and statistical inference were made using the Chi square test to assess risk factors and the t test to assess the degree of statistical significance when comparing means. Statistical significance was accepted at a 5% probability level, that is, a p-value of less than 0.05.

## Results

### Patient admission characteristics

Altogether a total of 2004 neonatal admissions from both hospitals were examined in this retrospective study. St Joseph’s Hospital reported 1,241 admissions, (62%), over the 3 year period including 290 in 2015, 455 in 2016 and 496 in 2017, whereas UWRH reported 763 neonatal admissions in 2017 alone accounting for almost two fifths (38%) of the total admissions (Table 1). Out of these, 1045 (52.1%) were male and 959 (47.9%) were female. The majority of patients, 1441 (71.9%) were admitted within the first day of birth which includes 89.3% of patients admitted to SJH and 46.3% of patients admitted to the UWRH (Table 2). There were also substantial admissions to UWRH within the 2^nd^ and 3^rd^day of birth, 185(24.3%), and between the 7^th^ to14^th^day of birth 159 (20.8%) (Table 2).

**Table 1:** Admissions and mortality of neonates at the UWRH & SJH.

	Number of admissions	Number of deaths
	N = 2004	N = 179
	n, %	n, %
Upper West Regional Hospital (2017)	763 (38.07)	68 (8.91)
St Joseph’s Hospital Jirapa 2017	496 (24.75)	30 (6.05)
St Joseph’s Hospital Jirapa 2016	455 (22.70)	48 (10.55)
St Joseph’s Hospital Jirapa 2015	290 (14.48)	33 (11.38)
St Joseph’s Hospital Jirapa 2015–2017	1241 (69.93)	111 (8.94)

**Table 2:** Age on admission at the UWRH & SJH.

Age at admission(days)	Overall	UWRH	SJH
	N = 2004	N = 763	N = 1241
	n, %^1^	n, %^1^	n, %^1^
≤ 1day	1441 (71.9)	333 (43.6)	1108 (89.3)
2–3 days	229 (11.4)	185(24.3)	44 (3.6)
4–6 days	128 (6.4)	86(11.3)	42 (3.4)
7–14 days	206 (10.3)	159(20.8)	47(3.8)

^1^ Column percentages

### Age at presentation

The mean age of presentation at UWRH was 4.4 days and 1.6 days at SJH (data were not shown). The differences in age at presentation on the first day between the two hospitals was statistically significant (p <0.001) (data were not shown).

### Gestational age and admission weight

About half 51.2% (391) of the admissions from the regional hospital were preterm and 48.8% (372) were born at term or beyond. Data on the actual gestational ages of the neonates were only available in 579 neonates from SJH out of which only 168 (29.1%) were preterm (Table 3). Additional data on the maturity of these neonates were available from the diagnosis given to some patients from SJH. Thus, combining the latter with the actual gestational ages increased the number of preterm neonates at SJH to 216 (22.1%) out of 979 neonates admitted to SJH.

**Table 3:** Gestation age distribution among infants admitted to UWRH and SJH.

	Total	UWRH	SJH
Gestational age (weeks)	N = 1342	N = 763	N = 579
	n, %	n, %	n, %
<28 weeks	19 (1.4)	14 (1.8)	5 (0.9)
28–29 weeks	104 (7.7)	89 (11.7)	15 (2.6)
30–33 weeks	134 (10.0)	98 (12.8)	36 (6.2)
34–36 weeks	302 (22.5)	190 (24.9)	112 (19.3)
37–42 weeks	771 (57.5)	368 (48.2)	403 (69.6)
>42 weeks	12 (0.9)	4 (0.5)	8 (1.4)

There were missing data on gestational age from SJH, 662 out of the 1241 (53.3%) patients admitted to SJH.

Information on the birth weights of the neonates was incomplete so admission weights have been presented in Table 4. The median weight on admission of the 763 patients from the UWRH was 2.8 Kg (inter quartile range (IQR) 2.1–3.3) and the median weight on admission of 1062 patients from SJH was 2.6 Kg (IQR 2.1–3.0). Table 5 depicts outcome by duration of stay and hospital. The duration of stay in hospital ranged from <24 hours to 66 days and most stayed for 3–6 days (59.4%) with a median duration of 3 days [IQR: 3–4 days] for UWRH and 5 days [IQR: 3–8 days] for SJH (Table 5). For neonates who died, the mean duration of stay for UWRH was 3.9 ± 3.1 and 4.9 ± 4.8 days for SJH (p = 0.097). In all, 26 out of 763 (3.4%) of patients admitted to UWRH died in the first 48 hours compared with 40 out of 1241 (3.2%) with no statistical difference between the two sites (p = 0.822).

**Table 4:** Outcome by admission weight and hospital.

		Outcome		
Hospital	Admission Weight (g)	Discharged	Died	Referred/absconded
		n, %^1^	n, %^1^	n, %^1^
	<1000; N = 7	3 (42.9)	4 (57.1)	-
	1000–1499; N = 58	41 (70.7)	17 (29.3)	-
**UWRH**	1500–1999; N = 93	81 (87.1)	12 (12.9)	-
	2000–2499; N = 131	124 (94.7)	7 (5.3)	-
	≥2500; N = 474	435 (91.8)	28 (5.9)	11 (2.3)
	**Total; N = 763**	**684 (89.7)**	**68 (8.9)**	**11 (1.4)**
	<1000; N = 7	4 (57.1)	3 (42.9)	-
	1000–1499; N = 56	40 (71.4)	15 (26.8)	1 (1.8)
**SJH**	1500–1999; N = 123	104 (84.6)	17 (13.8)	2 (1.6)
	2000–2499; N = 246	221 (89.8)	22 (8.9)	3 (1.2)
	≥2500; N = 630	593 (94.1)	33 (5.2)	4 (0.6)
	Total; N = 1062^*^	**962 (90.6)**	**90 (8.5)**	**10 (0.9)**

^1^Row percentages within each column; 179 missing records on admission weight for SJH

**Table 5:** Outcome by duration of stay and hospital.

		Outcome		
Hospital	Length of Hospital stay	Discharged	Died	Referred
		n, %^1^	n, %^1^	n, %^1^
		**N = 684**	**N = 68**	**N = 11**
	≤ 24 hrs	19 (2.8)	12 (17.7)	1 (9.1)
	>24–48 hrs	116 (17.0)	14 (20.6)	2 (18.2)
**UWRH**	3–6 days	479 (70.0)	31 (45.6)	7 (63.6)
	7–14 days	67 (9.8)	9 (13.2)	1 (9.1)
	>14 days	3 (0.4)	2 (2.9)	0 (0)
		**N = 1,119**	**N = 111**	**N = 11**
	≤ 24 hrs	55 (4.9)	20 (18.0)	4 (36.4)
	>24–48 hrs	79 (7.1)	20 (18.0)	3 (27.3)
**SJH**	3–6 days	620 (55.4)	47 (42.3)	3 (27.3)
	7–14 days	300 (26.8)	19 (17.1)	1 (9.1)
	>14 days	65 (5.8)	5 (4.5)	0 (0)
		**N = 1803**	**N = 179**	**N = 22**
	≤ 24 hrs	74 (4.1)	32 (17.9)	5 (22.7)
	>24–48 hrs	195 (10.8)	34 (19.0)	5 (22.7)
**All hospitals**	3–6 days	1,099 (60.4)	78 (43.6)	10 (45.5)
	7–14 days	367 (20.4)	28 (15.6)	2 (9.1)
	>14 days	68 (3.8)	7 (3.9)	0 (0)

^1^Column percentages within each super row

### Causes of admission

Infections alone accounted for 911(45.5%) of admissions and was the main diagnosis in almost a third (32.4%) of the patients who died (Table 6). Neonatal sepsis was the commonest cause of admission as well as the commonest infection occurring in 747(37.3%) neonates, followed by birth asphyxia in 303 (15.1%) and prematurity in 265(13.2%). Altogether 591(79.1%) of the neonates presented with early onset sepsis during the first three days after birth whereas 156(20.9%) presented with late onset sepsis after the third day of birth and beyond. A total of 529 (42.6%) of the neonates admitted to SJH were diagnosed with sepsis. Among those presenting with sepsis, 482 (91.1%) presented with early onset sepsis while 47(8.9%) presented with late onset sepsis at SJH. However, at UWRH, 218 (28.6%) of the neonates were diagnosed with sepsis out of which half, 109 (50.0%), presented with early onset sepsis and half 109(50.0%) presented with late onset sepsis.

**Table 6:** Main diagnosis of patients admitted and those who died at UWRH and SJH.

	Total	Outcome			
	N = 2004	Absconded	Discharged	Died	Referred
Cause of admission (Diagnosis)	n, %^1^	n, %^2^	n, %^2^	n, %^2^	n, %^2^
Neonatal Sepsis	**747 (37.3)**	2 (0.3)	692 (92.6)	47 (6.3)	6 (0.8)
Birth Asphyxia	**303 (15.1)**		266 (87.8)	37 (12.2)	
Prematurity	**265 (13.2)**		215 (81.1)	48 (18.1)	2 (0.8)
Low Birth Weight	**199 (9.9)**		185 (93.0)	12 (6.0)	2 (1.0)
Neonatal Jaundice	**155 (7.7)**		141 (91.0)	12 (7.7)	2 (1.3)
Pneumonia	**42 (2.1)**		36 (85.7)	6 (14.3)	
Cord Sepsis	**38 (1.9)**		37 (97.4)	1 (2.6)	
Macrosomic baby	**36 (1.8)**		35 (97.2)	1 (2.8)	
Impetigo	**31 (1.5)**		29 (93.5)	1 (3.2)	1 (3.2)
Congenital Malformation	**25 (1.2)**	1 (4.0)	20 (80.0)	2 (8.0)	2 (8.0)
Others	**24 (1.2)**		22 (91.7)	1 (4.2)	1 (4.2)
Meconium aspiration	**23 (1.1)**		21 (91.3)	2 (8.7)	
Anaemia	**20 (1.0)**		18 (90.0)	1 (5.0)	1 (5.0)
Bleeding	**17 (0.8)**		17 (100)		
Ophthalmianeonatorium	**16 (0.8)**		14 (87.5)	2 (12.5)	
Malaria	**13 (0.6)**		13 (100)		
Other Infection	**12 (0.6)**		12 (100)		
*Birth Injury	**7 (0.3)**		4 (57.1)	1 (14.3)	2 (28.6)
Hypoglycaemia	**7 (0.3)**		5 (71.4)	2 (28.6)	
Malnutrition	**7 (0.3)**		7 (100)		
Gastroenteritis	**6 (0.3)**		6 (100)		
RTI	**6 (0.3)**		5 (83.3)	1 (16.7)	
Unspecified	**2 (0.1)**		2 (100)		
URT Obstruction	**2 (0.1)**			2 (100)	
Cardiac Arrest	**1 (0.1)**		1 (100)		

^1^% are column percentages

^2^% are row percentages within each cause of admission; RTI—Respiratory Tract Infection; URT–Upper Respiratory Tract

*The 7 babies with birth injuries included 2 with fractured femur; 3 unspecified labeled as palsy, trauma and birth injury respectively; and two babies with soft tissue injuries comprising facial bruises and a swollen penis

Low birth weight was reported as the main or sole diagnosis in 199(9.9%) patients, and neonatal jaundice in 155 (7.7%). These are presented in Table 6. Out of the 13 patients with malaria, eight (8) were reported to be congenital, four (4) patients were born premature, and three (3) including two (2) with congenital malaria were low birth weight. Out of a total of 303 neonates with birth asphyxia, 177 (14.3%) of total admissions were from SJH whereas 126 (representing 16.5% of total admissions) were from UWRH.

### Mortality

Altogether, 179 (8.93%) neonates died. The overall proportion of neonatal deaths was similar in both hospitals with a proportion of 8.94% at the SJH and 8.91% at UWRH (p = 0.980) and has been displayed in Table 1. The difference between the proportion of deaths in 2017 at SJH (6.05%) and UWRH (8.91%) was also not statistically significant (p = 0.064). Of those who died, prematurity was the commonest cause of death, (26.8%), followed by neonatal sepsis, (26.3%), birth asphyxia, (20.7%), low birth weight, (6.7%), neonatal jaundice, (6.7%) and pneumonia, (3.4%) as shown in Table 6. Mortality was significantly associated with being low birth weight, premature and being less than three days old at presentation and staying in hospital for a duration of 48 hours or less (Table 7). A total of 66 neonates (36.9%) died within 48 hours after admission but only 279 (15.3%) were discharged or referred within this period. The majority, 113 (63.1%) neonates died after 48 hours and most of deaths, 43.6% (78) occurred in those who spent between 3–6 days in both hospitals, however, they were also responsible for majority, 1,109(60.8%) of the patients who were discharged and referred Table 5. Case fatality was high for neonates with upper respiratory tract obstruction (100%), hypoglycaemia (23.6%), prematurity (18.1%), respiratory tract infection (16.7%), pneumonia (14.3%), birth injury (14.3%), ophthalmia neonatorum (12.5%) and birth asphyxia (12.2%) as shown in Table 6. Admission weights for the hypoglycaemic patients ranged from 2.1 to 3.3 kgs.

**Table 7:** Factors associated with neonatal mortality at the UWRH & SJH.

		Outcome			
Characteristic		Died	Alive	Odds ratio [95% CI]	p-value
		n, %	n, %		
Sex					
	Male	100 (55.9)	945 (51.8)	1.04 [0.87–1.61]	0.297
	Female	79 (44.1)	880 (48.2)	1.00	
Duration of stay					
	≤ 48 hours	66 (36.9)	279 (15.3)	3.24 [2.33–4.50]	**<0.001**
	>48 hours	113 (63.1)	1,546 (84.7)	1.0	
Weight					
	<2.5 Kg	97 (61.4)	624 (37.4)	2.66 [1.90–3.72]	**<0.001**
	≥2.5 Kg	61 (38.6)	1,043 (62.6)	1.0	
Gestational age					
	<37 weeks	61 (52.1)	498 (40.6)	1.59 [1.09–2.34]	**0.017**
	>42 weeks	1 (0.9)	11 (0.9)	1.18 [0.15–9.34]	0.873
	37–42 weeks	55 (47.0)	716 (58.5)	1.0	
Age					
	<72 hours	155 (86.6)	1,396 (76.4)	1.98 [1.27–3.09]	**0.002**
	≥72 hours	24 (13.4)	429 (23.5)	1.0	

## Discussion

This study highlights some differences and similarities between patients seen at a district hospital with a well-established neonatal unit which has been operating for three (3) years and a recently established neonatal unit in a regional hospital which has been operating for a year. The district hospital differed from the regional hospital by having fewer admissions and over 80% of its admissions presenting within the first day of birth compared to 43.6% who presented on the first day at the UWRH. The former is similar to a study which reported the average age of neonates at admission to be 1.57days [13]. Although the actual numbers of inborn and outborns in this study is unknown, the observed difference between the hospitals in our study is possibly because most of the patients from the SJH were likely to be inborn as UWRH receives more referrals from the rest of the region. These referring units may have spent time trying to stabilize the neonates before the referral and is supported by a study at a tertiary neonatal unit which had a mean age of outborn admissions at 3.13 days compared with a mean for inborn admissions at <1 day [13]. Also being a well-established facility with a neonatal unit, in-utero transfers to SJH are more likely. There was an increase in admissions from day 7–14 of birth at the UWRH. This may have been due to late onset neonatal sepsis which was more prevalent at the UWRH. It could also have been a reflection of the cultural belief that babies must be kept indoors for 1–6 weeks in some communities in the Upper West Region and must not travel, delaying health interventions [14].We also noted that there were fewer patients staying for 48 hours or less at SJH and a longer mean duration of stay but the reason for this is uncertain.

The proportion of neonates who died at the UWRH in 2017 was 8.91% which was more than those who died at SJH in 2017(6.05%). The mortality trend at SJH also showed a consistent declining trend from 2015 and 2017. However, the overall proportion of deaths was similar in both hospitals with 8.94% at SJH and 8.91 at UWRH with no significant difference between the two (p = 0.980). This finding is comparable to other studies in similar settings; a regional hospital in Cameroon recorded 9.83%, a tertiary care centre in India reported 10.4%, two regional provincial hospitals in Laos reported 8.9% and 10% respectively [6, 15, 16]. The observation was however higher than the 1% reported by a district hospital in India and 2.3%and 6.8% reported by two of the regional provincial hospitals in Laos and the 6.0% reported at SJH in 2017 in this study [16,17]. However, it was lower than the 15.7% reported by a regional hospital in Cameroon, 11.3%, reported by a tertiary hospital in Tanzania and 13.3% reported by a referral hospital in Ethiopia [18–20]. It is also lower than the mortality reported by two teaching hospitals in Nigeria which reported 14.2% and 19.4% respectively and a teaching hospital in Mozambique and Ghana which reported 18% and 18.8% respectively [13,21–23]. The higher numbers reported by the latter may be due to more high risk admissions to these facilities. Similarly, one would have expected a higher mortality at the regional hospital due to the referral of high risk babies there which was portrayed when comparing the proportions by year. Further studies on outcomes from neonatal units in low- and middle-income countries are required to provide comprehensive bench marks for evaluating care with consideration to available facilities, staffing and skills.

Neonatal sepsis, prematurity, birth asphyxia, low birth weight neonatal jaundice and pneumonia were the most common reasons for admission and causes of morbidity in this study. This pattern of disease is similar to reports from studies in Laos and a rural tertiary Nigerian [16, 24].However it differs in sequence from the study in Nigeria, India and Tanzania which ranked birth asphyxia as the most common cause of admission followed by prematurity or low birth weight [13, 15, 19]. The findings also differs from a study in India which reported hyperbilirubinemia, birth asphyxia/trauma, sepsis and respiratory distress due to transient tachypnoea of the newborn, meconium aspiration syndrome, pneumonia and hyaline membrane disease as the commonest diseases and a study in a University hospital in Ethiopia which reported hypothermia, neonatal sepsis, polycythaemia, hypoglycemia, perinatal asphyxia, hyperbilirubinemia, respiratory distress syndrome to be most common [17, 25]. These differences may be partly related to the level of sophistication of the service. Prematurity, neonatal sepsis, birth asphyxia, neonatal jaundice, and low birth weight were the most common conditions reported among the patients who died and this findings are similar to that of a studies from Kumasi, Ghana, Cameroun and Nigeria [5, 6, 13].

The diagnosis of infectious disease in almost half (45.5%), of the patients suggests that majority of diseases in the newborns in this setting can be prevented and is similar to reports from Laos which reported 44% and 76% in two of its hospitals [16]. The diagnosis of sepsis was mainly clinical and accounted for over a quarter (26.3%) of the deaths. There was high prevalence of early onset sepsis in SJH and equal proportions of early and late-onset sepsis at UWRH. Early onset sepsis (EOS) occurs within 72 hours of birth and late on sepsis (LOS) occurs after 72 hours. EOS is usually associated with ascending infection from the genital tract and infection from the placenta while LOS is often from environmental sources such as nosocomial or community acquired infection [26,27]. This description provides additional information on possible causes of infection to aid control. However, better diagnostics are required to exclude antimicrobial resistance, other diseases and to monitor the pattern of cross infection between community acquired and nosocomial infection so that changes to infection control and antibiotic protocols can be made periodically as necessary [13]. A reduction in the cases of neonatal sepsis can be achieved through clean delivery practices, intrapartum antibiotics for maternal Group B streptococcus, hand washing by caregivers and health professionals, early initiation of breastfeeding, maternal and other forms of neonatal skin antisepsis [8, 27, 28,29].These measures can reduce the work load, shorten the duration of stay, avoid cross infection and reduce poor outcomes [30]. Newer interventions involving the use of immunomodulators such as lactoferrin, prebiotics, probiotics, synbiotics and pentoxifylline (ptx), a non- phosphodiesterase inhibitor can be introduced [31].

Respiratory infection and pneumonia were associated with high case fatality. This has been observed in other studies and it highlights the need to prevent pneumonia, respiratory infection and support respiration [8, 28]. Early recognition and referral when the signs of respiratory distress occur need to be promoted [32]. The two patients who presented with upper respiratory tract obstruction did not survive and it probably highlights the need for more specialised care and respiratory support as they may have been associated with congenital malformations [33]. The high prevalence of neonatal jaundice may be related to sepsis as well as Glucose-6-Dehydrogenase deficiency which has an estimated prevalence of 15–26% in the Ghanaian population [34].This may have been aggravated by late presentation resulting from long distances travelled and inappropriate health seeking behaviour which are not uncommon in this setting [1, 8, 35, 36]. Screening for these conditions is therefore necessary [37].

Two of the patients who presented with ophthalmia neonatorum did not survive. Although ophthalmia neonatorum is most commonly due to Neisseria gonococcus and Chlamydia, in recent times, Mycoplasma genitalium has also been reported to be an important cause of the disease [38–40]. While gonococcus may have caused disseminated infection leading to the demise of these patients, the pneumonitis caused by Chlamydia infection can do the same though it usually presents at 3–6 weeks and beyond, thus the possibility of antibiotic resistance also needs to be considered [38–40]. Confirmatory tests for these diseases are unavailable in this and similar setting thus screening and treating sexually transmitted infection during the antenatal period using the syndromic approach may reduce the condition [38]. Gonococcal and Chlamydia disease can be prevented by giving all newborns, prophylactic antibiotic eye drops such as Erythromycin and Tetracycline eye drops or Povidine Iodine eye drops. However, implementation barriers need to be overcome [39,40]. Although silver nitrate has been used in the past, it is no longer recommended and the use of a fatty acid-based formulation is currently being investigated [40,41].

Reducing infection will also reduce mortality from prematurity, the commonest cause of neonatal deaths in this study [8]. Malaria was reported in 13 patients. Though it is reassuring to note that none of these patients died, its association with prematurity and low birth weight are well known and studies have shown that it is more common than previously reported with similar presentation as neonatal sepsis or jaundice [42, 43].Recent evidence suggests that the effect of malaria on pregnant women may be strain specific with those infected with the 3D7-like variants of VAR2CSA in *P*. *falciparum*, having the worst outcome including low birth weight [44]. Thus since the area is one of the most highly malaria endemic regions in the country, it will be expedient to screen patients with sepsis for malaria and investigate the contribution of malaria to prematurity and low birth weight as well as the uptake of malaria preventive interventions in pregnancy. Further reductions in deaths from prematurity may require improved antenatal care to prevent the condition and upgrading current services to include more specialized care but this is resource intensive [8].

The contribution of birth asphyxia to mortality in this study is not an unexpected finding since several risk factors for perinatal mortality have been identified in this setting [35,36]. Apart from the severity of the neonatal conditions themselves, health service factors affecting the smooth running of the neonatal units might also have militated against neonatal survival. These include shortages of materials, lack of a paediatrician, power outages barring electricity supply to the incubators which necessitated the purchase of energy storage devices and deficiencies in the quality of inter-facility transport and primary care, among others [45,46,47]. Reducing these factors require interventions within and beyond the health sector and must include multi-sectoral actions and political will tied with economic development. Patients admitted with a diagnosis of hypoglycaemia had a high case fatality rate. Hypoglycaemia is common in preterm, small for gestational age babies, infants of diabetic mothers, and in metabolic or hormonal disease [48]. The causes need to be identified as it can cause irreversible brain damage thus aggressive screening and treatment is recommended. Congenital malformations and bleeding did not appear to be a major cause of mortality in this setting, similar to reports from other studies [15, 17, 49]. However, two patients with congenital malformations required transfer for further management. Additional effort to avoid birth injuries is needed since one patient died and the two with fractured femurs were referred to a tertiary unit. Birth injuries have also been reported to be an important cause of neonatal mortality in the Northern Region which shares a border with this region [50].

Altogether a total of 19 patients were referred for specialist care. Some of these babies would have been sent to Komfo Anokye Teaching Hospital in Kumasi which is 445km away or an average of 8 hours’ drive or the Teaching Hospital of the University of Development studies in Tamale which is 303kms away or about 5 hours drive from the UWRH. These distances might also have influenced the decision to transfer patients especially if the patients are unstable or impoverished as they have to make out-of-pocket payments for the National Ambulance Transport services and it could have impacted on the mortality. Having a paediatrician or neonatologist to drive training would reduce the need for transfers especially at the Regional Hospital which has a mandate to provide specialist care. The presence of paediatricians has been reported to influence child mortality. [51,52].

Additionally, specialists such as paediatric surgeons and paediatric anaesthesiologist at the regional hospital can reduce the need for transfers to deal with congenital malformations, injuries and other surgical conditions such as necrotising enterocolitis. A review of some perinatal deaths occurring in 2017 and 2018 showed rapid deterioration of surgical patients which was striking and they became unstable for transfer; we also noted their prominence among outborns, some of whom would have travelled long distances to get to UWRH [47]. Furthermore, a study from Tamale Teaching Hospital showed that mortality among neonatal surgical patients referred from the Upper West Region was worse than its patients [53]. However, there are arguments for and against decentralizing these services particularly when patient numbers are low so critical evidence is needed [33,54].Studies have also shown that some patients transferred to tertiary hospitals in India, Bangladesh, Cameroon and Nigeria had hypothermia and hypoglycaemia on arrival as well as higher mortality [55–58]. Further evidence to support the case for full-time or visiting specialists at the regional level and the use of retrieval teams for stabilizing neonates before and during transfer is needed since in-utero transfer though preferred, is not always possible [58–61].

We encountered some missing data especially on gestational age and birth weight which may have influenced the proportions of patients in the gestational age categories. These indicators are important for managing preterm babies and evaluating care and can be obtained by applying the Ballard scoring system to all admitted babies and using an admission form to enable the referring units to provide this information on admission. The latter was implemented by SJH during the data collection. We were unable to obtain information on inborn and outborn infants which would have yielded useful additional information. The cases of sepsis were not confirmed with blood culture and antimicrobial sensitivity testing and therefore are suspected cases of sepsis. It is possible that some of the cases may have been wrongly labeled as neonatal sepsis, however the relatively low overall mortality compared with other studies and high discharge rate in these patients suggests a positive response to empiric treatment with antibiotics and the likelihood of actual infection. We have related mortality to admission diagnosis which may differ from the actual cause of death due to lack of information and it may be due to multiple causes. Lack of explicit data on diagnostic criteria also prevented us from linking conditions such as kernicterus to mortality from neonatal jaundice. We compared 3-year data from SJH with one year from UWRH as these were the data available. We studied neonatal mortality for one year in UWRH and 3 years at SJH to allow for future comparisons between regions and districts. Though it seems incongruous, we compared mortality in 2017 at both hospitals to mitigate this effect and collected the data from the neonatal unit at UWRH, 5 months after it was started to allow time for impact. We did not provide information on treatments received which could also have influenced outcome thus further studies are needed in this area.

## Conclusion

In spite of having a more recent neonatal unit, more admissions and high risk babies at the UWRH, the overall proportions of neonatal deaths were similar in both hospitals, because data were collected for only 2017 for UWRH. However, it compares favourably with the lower end of outcomes in similar settings. More attention needs to be placed on better collection of routine data such as birth weight and gestation age. Further studies on risk factors for sepsis, prematurity and low birth weight and the contribution of malaria to these conditions are needed. In addition, studies to determine the epidemiology of neonatal sepsis, the causative organisms, their sensitivity patterns to antimicrobials and cross infectivity patterns are desirable. In order to improve outcome, more skills at stabilising neonates in the first few days of admission are required including respiratory support. In addition, multi-sectoral actions and political will are required to prevent birth asphyxia and achieve the Sustainable Development Goal to end preventable deaths of newborns and reduce neonatal mortality in this and similar settings.

## Supporting information

S1 Data (XLSX)
